# Novel iron chelator SK4 demonstrates cytotoxicity in a range of tumour derived cell lines

**DOI:** 10.3389/fmolb.2022.1005092

**Published:** 2022-09-23

**Authors:** Gina Abdelaal, Andrew Carter, Mihalis I. Panayiotides, David Tetard, Stephany Veuger

**Affiliations:** ^1^ Department of Applied Sciences, Faculty of Health and Life Sciences, Northumbria University, Newcastle upon Tyne, United Kingdom; ^2^ Department of Cancer Genetics, Therapeutics and Ultrastructural Pathology, The Cyprus Institute of Neurology and Genetics, Nicosia, Cyprus

**Keywords:** iron, iron chelation, LAT1, NDRG1, cancer, drug design, SK4

## Abstract

Iron is an essential micronutrient due to its involvement in many cellular processes including DNA replication and OXPHOS. Tumors overexpress iron metabolism linked proteins which allow for iron accumulation driving high levels of proliferation. Our group has designed novel iron chelator SK4 which targets cancer’s “iron addiction.” SK4 comprises of two key moieties: an iron chelation moiety responsible for cytotoxicity and an amino acid moiety which allows entry through amino acid transporter LAT1. We selected LAT1 as a route of entry as it is commonly overexpressed in malignant tumors. SK4 has previously demonstrated promising results in an *in vitro* model for melanoma. We hypothesized SK4 would be effective against a range of tumor types. We have screened a panel of tumor-derived cell lines from different origins including breast, prostate, ovarian and cervical cancer for SK4 sensitivity and we have found a range of differential sensitivities varying from 111.3 to >500 μM. We validated the iron chelation moiety as responsible for inducing cytotoxicity through control compounds; each lacking a key moiety. Following the screen, we conducted a series of assays to elucidate the mechanism of action behind SK4 cytotoxicity. SK4 was shown to induce apoptosis in triple negative breast cancer cell line MDA MB 231 but not ovarian cancer cell line SKOV3 suggesting SK4 may induce different modes of cell death in each cell line. As MDA MB 231 cells harbor a mutation in p53, we conclude SK4 is capable of inducing apoptosis in a p53-independent manner. SK4 upregulated NDRG1 expression in MDA MB 231 and SKOV3 cells. Interestingly, knockdown of NDRG1 antagonized SK4 in MDA MB 231 cells but not SKOV3 cells suggesting SK4’s mechanism of action may be mediated through NDRG1 in MDA MB 231 cells. In conclusion, we have shown tagging iron chelators with an amino acid moiety to allow entry through the LAT1 transporter represents a double pronged approach to cancer therapy, targeting “iron addiction” and amino acid metabolism dysregulation.

## 1 Introduction

Iron is an essential dietary micronutrient and one of the most common dietary deficiencies (McLean et al., 2009; Stevens et al., 2013). Approximately 2% of human proteins are bound to iron in the form of haem, iron sulfur clusters or as individual iron ions (Andreini et al., 2018). Iron is a cofactor for many enzymes involved in cell survival and metabolism. For example, ribonucleotide reductase—the enzyme which synthesizes the building blocks for DNA synthesis—relies on iron as a cofactor (Mulliez et al., 1999; Li et al., 2017). Additionally, iron supports cellular metabolism through its role in the mitochondrial iron-sulfur cluster providing cells with their energy requirements (Singer and Johnson, 1985). In conclusion, normal cellular behaviors are dependent on iron.

Iron can transform from one oxidative state to another through the Fenton reaction producing reactive oxygen species in the process, inducing oxidative stress in cells, leading to DNA damage and eventually malignant mutations (Winterbourn, 1995; Linn, 1998; Torti and Torti, 2020). Epidemiological studies and animal models have shown an excess of iron can increase the risk of cancer. Mice with a higher intake of dietary iron suffered from larger tumors than their counterparts who were fed a lower iron diet (Hann et al., 1991; Thompson et al., 1991). Additionally, tumors ranging from breast, prostate and colorectal cancers have an abnormal balance in iron metabolism proteins favoring pro-iron proteins such as transferrin: an iron importer (Cui et al., 2007; Zhang et al., 2020; Wei et al., 2021). High levels of iron exporter ferroportin have been associated with positive patient prognosis in breast cancer (Pinnix et al., 2010). All in all, studies show an excess of iron and aberrant iron metabolism can drive cancer development and progression.

The reliance of cancer on iron for survival has led to the proposal of “iron addiction” as a target for anticancer therapy. Iron chelator drugs drive cytotoxicity through stripping cancer cells of their iron supply. *In vitro* studies have shown iron chelation to be effective against a range of tumor-derived cell lines (Whitnall et al., 2006). Iron chelation is capable of inducing cytotoxicity in p53 deficient cell lines and stem cancer cell lines (Whitnall et al., 2006; Katsura et al., 2019). The most well-studied iron chelators are DFO and Dp44mT. DFO was initially used for iron overload treatment; work in cell lines supported anticancer activity (Lui et al., 2015a). Dp44mT has been successful *in vivo*, with xenograft mice treated with Dp44mT had a decrease in tumor size and no change in hematological indices (Whitnall et al., 2006). N-myc downstream-regulated gene 1 (NDRG1) has been implicated in the underlying iron chelation mechanism of action. Treatment of cell lines and mouse models with iron chelation upregulates NDRG1 expression driving cell signaling changes mainly through inhibition of oncogenic cell signaling pathways such as EGFR, TGF-β, and STAT3 (Le and Richardson, 2004; Lui et al., 2015b; Menezes et al., 2019; Yang et al., 2021). We have recently published a review article highlighting iron chelation as capable of reversing several hallmarks of cancer through NDRG1 disrupting oncogenic signaling pathways (Abdelaal and Veuger, 2021). Despite promising work *in vitro* and *in vivo* iron chelation has not yielded positive results in clinical trials indicating more work needed to develop effective iron chelation treatment.

**FIGURE 1:**
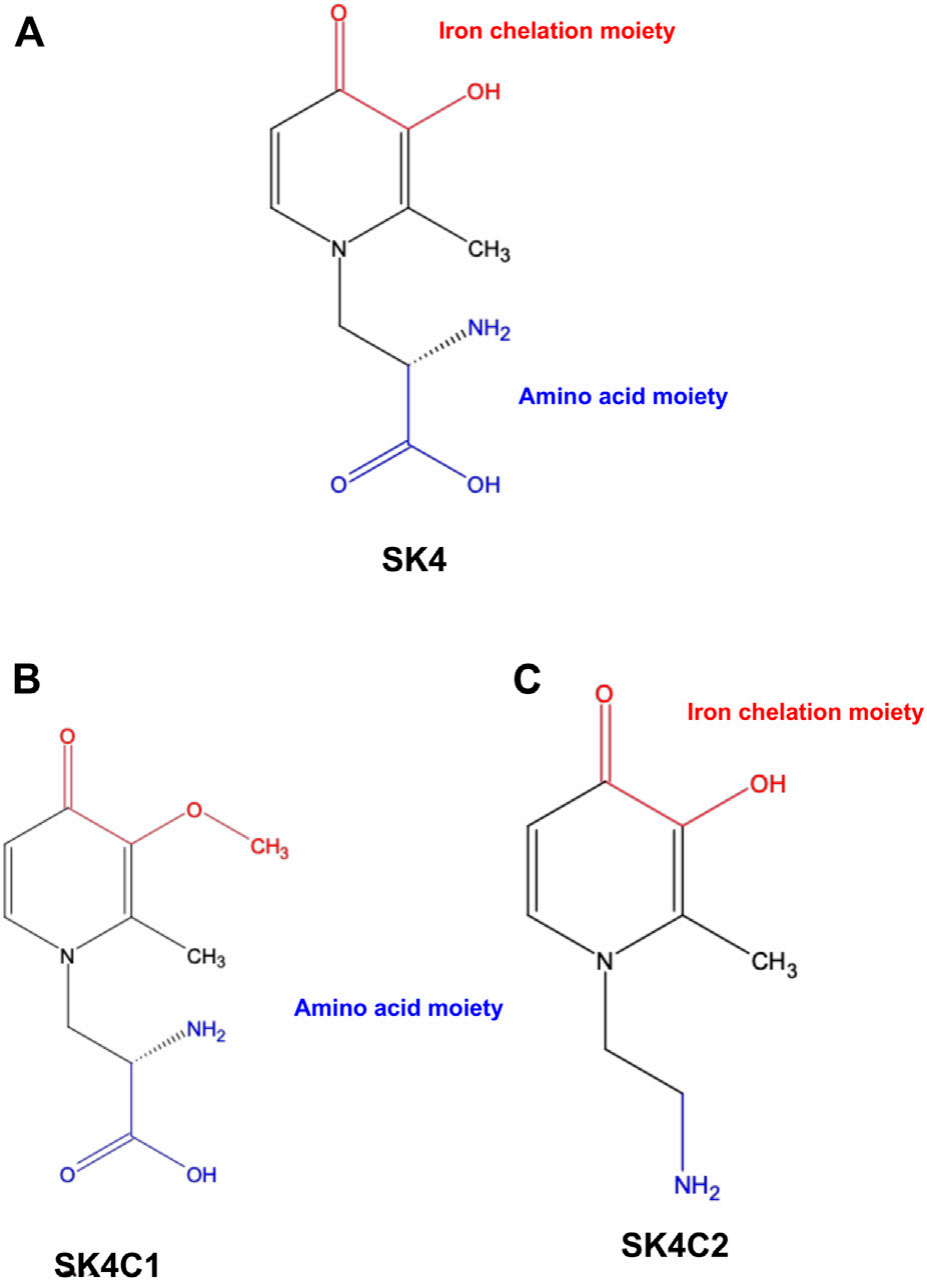
Chemical structures. SK4 **(A)** consists of two key moieties: An iron chelation moiety and an amino acid moiety to allow entry through the LAT1 transporter. Control compound SK4C1 **(B)** lacks the iron chelation moiety while SK4C2 **(C)** lacks the amino acid moiety.

Our group has designed a novel iron chelator SK4; formed of two moieties: A moiety responsible for iron chelation and a moiety responsible for entry through the L-type amino acid transporter (LAT1) transporter (Figure 1). SK4 is a methylated analogue of L-mimosine, which has previously demonstrated its iron chelation capabilities via metal binding studies. Additionally, SK4 has been shown to inhibit erastin mediated ferroptosis in an *in vitro* model for Parkinson’s disease (Gutbier et al., 2020; Kyriakou et al., 2020; Kyriakou et al., 2021).

We selected LAT1 as the mode of cell entry as LAT1 is commonly overexpressed in cancer including breast, prostate and ovarian (Sakata et al., 2009; Segawa et al., 2013; Kaira et al., 2015; Ichinoe et al., 2021; Kurozumi et al., 2022). Additionally, metastatic tumors tend to have higher levels of LAT1 (Ichinoe et al., 2015; Papin-Michault et al., 2016). In contrast, more conventional iron chelators DFO and Dp44mT gain cell entry through the plasma cell membrane, with DFO demonstrating poor lipophilicity and as a result limited cell entry whilst Dp44mT is highly lipophilic which could result in side effects.

SK4 has shown promising results in melanoma cell lines and induced cell cycle arrest and apoptosis (Kyriakou et al., 2020; Kyriakou et al., 2021). We hypothesized that SK4 would be effective against a range of cancers, as “iron addiction” is a universal cancer trait. We conducted a screen against a panel of cells lines and observed differential sensitivity, as well differences in apoptosis and cell cycle distribution in response to treatment. We also investigated the involvement of NDRG1 in SK4 mediated cytotoxicity.

## 2 Materials and methods

### 2.1 Cell culture

Human dermal fibroblasts (HDF) (Gift from Dr. Stephan O’Reily), HeLa cells (Gift from Dr. Antony Antoniou), PC3 (Gift from Dr. Luke Gaughan), DU145 (Gift from Dr. Luke Gaughan), SKOV3 (Sigma Aldrich), MDA MB 231 (Sigma Aldrich) cell lines were used in this study. MDA MB 231, HeLa and HDF cell lines were cultured routinely in Dulbecco’s modified Eagle’s medium (DMEM), PC3 and DU145 cell lines were cultured routinely in Rosewell Park Memorial Institute (RPMI 1640), and SKOV3 cell lines were cultured routinely in Mcoy’s media (Gibco). Cell culture media was supplemented with 10% fetal bovine serum (FBS) and 1% penicillin/streptomycin (Gibco). Number of passages was limited to 20 and the cell lines were routinely tested for mycoplasma.

### 2.2 siRNA knockdown treatments

Cells were seeded in six well plates at 2 × 10^5^/well and transfected with control siRNA (Dharmacon) or NDRG1 siRNA (Thermofisher, Massachusetts) with HiPerFect transfection reagent (Qiagen, Germany) according to manufacturer’s recommendation. Cells were incubated with siRNA for 6 h then media was removed and replaced with fresh media or media spiked with drug.

### 2.3 Cell viability assays

SK4 utilized in this study was made in house (Kyriakou et al., 2020). Cell viability was evaluated through sulforhodamine B (SRB) assay. Cells were grown in 96 well plates at 2,000–5,000 cells/well and treated with increasing concentrations of SK4: 10, 50, 100, 250, and 500 µM. Cells were fixed in Carnoy’s solution (3:1 methanol to acetic acid) for 1 h or overnight at 4°C. Plates were washed five times in dH_2_O, then stained for 30 min with 0.03% SRB dye. Excess SRB dye was washed off using 1% acetic acid and the SRB dye was solubilized with 10 mM Tris base pH 10 for 30 min on a plate rocker. Plates were visualized on the FLUOstar^®^ Omega Plate reader (BMG Labtech, Bucks). Data was analyzed through Graphpad Prism version 9.0 through four parameter non-linear regression. Results were displayed as normalized response percentage of the control. As a confirmation of SRB assay results, trypan blue staining and cell counting on a haemocytometer slide was used.

### 2.4 Western blotting

Cells were grown in six well plates and treated with their respective GI50/IC50 of SK4 for a time course. Cells were scraped off using 1X RIPA buffer (10 mM Tris-HCl pH 8.0, 1 mM EDTA, 1% Triton X-100, 0.1% sodium deoxycholate, 0.1% SDS, 140 mM NaCl) supplemented with Halt™ Protease inhibitor Cocktail (Thermofisher, Massachusetts), United States and Halt™ Phosphotase Inhibitor Cocktail inhibitors (Thermofisher, Massachusetts) and cell lysates were assessed for protein concentration using a bicinchoninic acid (BCA assay) (Thermofisher, Massachusetts). Samples were incubated in 6X SDS loading buffer (6% SDS, 4.8% glycerol, 9% 2-mercaptoethanol, 2% bromophenol blue and 375 mM Tris HCl, pH 6.8) at 65°C for 10 min then loaded onto 4%–20% Mini-PROTEAN^®^ TGX™ Precast Protein Gels (Biorad, California) and electrophoresed at 100 V until the dye front reached the bottom of the tank. Proteins were transferred to Polyvinylidene fluoride membrane at 400 mA for 1 h and 30 min. Membranes were blocked with 2% milk in 1XTBST (20 mM Tris, pH 7.5 150 mM NaCl pH 7.6) and then incubated overnight with primary antibodies: rabbit NDRG1 [Abcam, (Cambridge) # ab124689; 1 in 10,000], PARP-1 {Cell signaling, [Cell Signaling Technology (Massachusetts) # 9542; 1 in 1000]}, and GAPDH [Abcam, (Cambridge) # ab181603; 1 in 10,000]. The membranes were incubated in Goat anti-rabbit HRP conjugated secondary antibodies [Dako (Cambridgeshire) #P0448; 1 in 1000] for 1 h and the SuperSignal™ West Dura Extended Duration Substrate (Thermofisher scientific, Massachusetts) was added for 1 min in the dark. Membranes were analyzed with the G:Box chemi XX6 imaging system (SynGene, Cambridge). ImageJ was used for densitometry analysis.

### 2.5 Immunofluorescence

Cells were grown on 12 mm coverslips in 24 well plates and treated with their respective GI50/IC50. Cells were fixed with 4% paraformaldehyde (Thermofisher, Massachusetts) for 20 min then washed three times with 1X PBS. Coverslips were blocked in 1% BSA/0.03% triton-X/1X PBS for 1 h then incubated in NDRG1 primary antibody [Abcam, (Cambridge) #ab124689; 1 in 10,000] overnight at 4°C. Coverslips were incubated in Anti-rabbit Alexafluor 568 conjugated secondary antibody (Invitrogen #1134929; 1 in 1000) for 1 h then mounted onto slides using Fluoromount-G™, Mounting Media with DAPI (Thermofisher, Massachusetts). Slides were visualized using the Leica DMi8 inverted confocal microscope (Leica Biosystems, Germany).

### 2.6 Cell cycle analysis

Cells were grown in six well plates and treated with their respective GI50/IC50. Cells were harvested and fixed in 70% ethanol for 1 h at 4°C. Cell pellets were washed twice in 1X PBS then centrifuged at 900 g for 3 min. Cell pellets were treated with 100 μg/ml RNAse A for 20 min at 37°C. Propidium iodide (Invitrogen) was added at 50 μg/ml and the cell suspensions were analyzed through the FACS Canto II flow cytometer (BD Biosciences, California) and FlowJo™ v10.8 Software (BD Life Sciences).

### 2.7 Annexin V assay

Cells were grown in six well plates and treated with their respective GI50/IC50. To investigate apoptosis cells were harvested and the eBioscience™ Annexin V Apoptosis Detection Kit APC kit was used according to manufacturer’s recommendation. Cells were harvested, washed once in 1X PBS, and once in 1X Binding buffer. Cell pellets were resuspended in 1X Binding buffer and 5 µl of Annexin-APC was added for 15 min at room temperature in the dark. Cells were washed once more in 1X Binding buffer and 5 µl of propidium iodide was added. Cell suspensions were analyzed through the FACS Canto II flow cytometer (BD Biosciences, California) and FlowJo™ v10.8 Software (BD Life Sciences).

### 2.8 Scratch wound assays

Cells were grown in six well plates and allowed to reach 70%–90% confluence. Scratches were made across the wells with a p200 pipette tip, media was removed and replaced with fresh media or fresh media spiked with drug at respective IC50/GI50 concentrations. Scratches were imaged at 0, 24, 48 and 72 h with the Leica DMi1 cell culture light microscope. Images were analysed through ImageJ software.

### 2.9 Statistical analysis

Graphpad Prism version 9.0 was used for statistical analysis. Error bars are representative of the three independent biological replicates means ± the standard error. Significance was calculated through an unpaired Student’s *t*-test for comparisons between control and treated cells. For multiple group comparisons data were analyzed through a one-way ANOVA followed by a Bonferroni post hoc test.

## 3 Results

### 3.1 Screening the panel of cell lines

**FIGURE 2:**
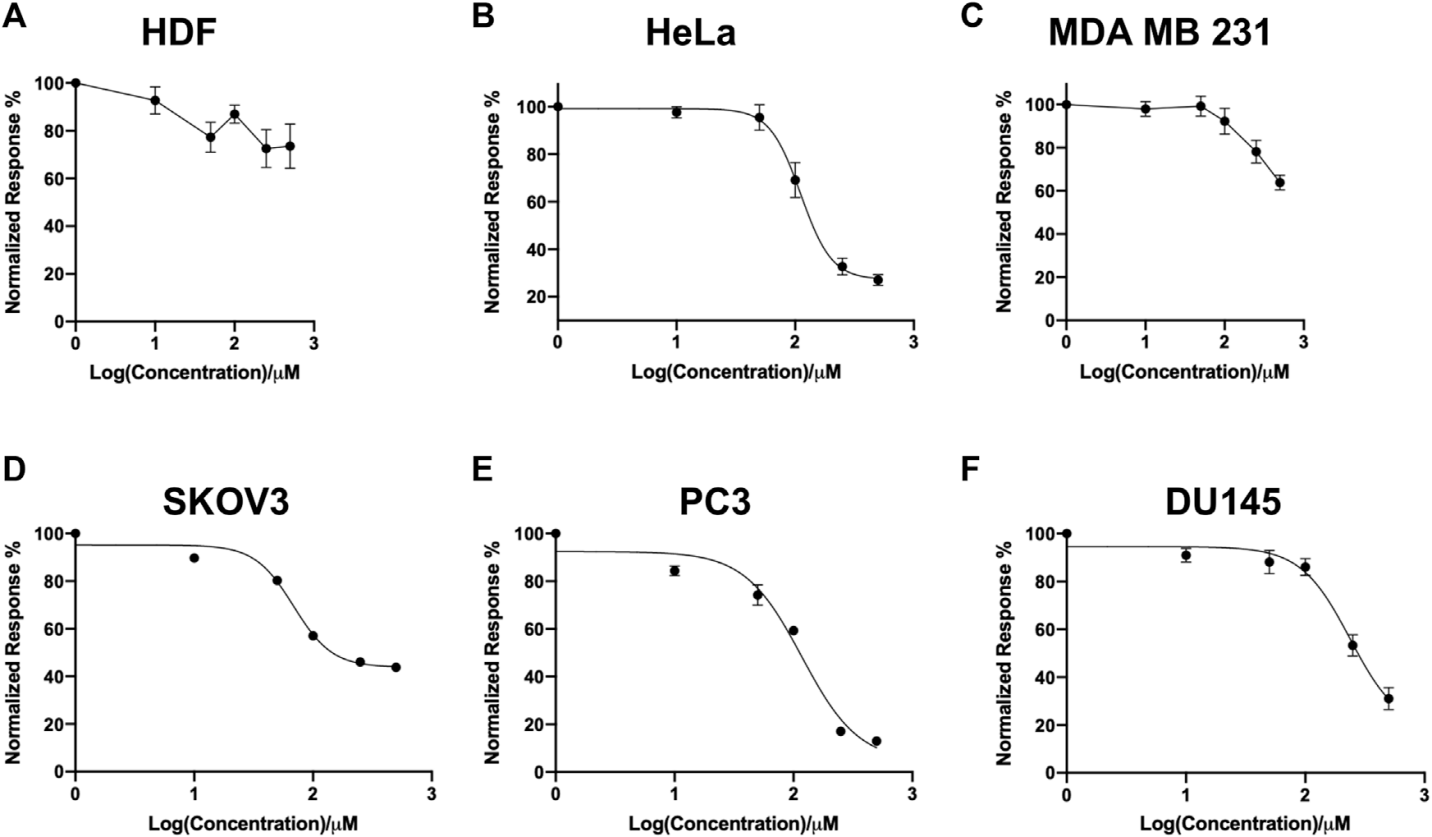
Screen of cell line panel reveals differential sensitivity to SK4. **(A)** Human dermal fibroblasts, **(B)** cervical cancer HeLa, **(C)** breast cancer MDA MB 231, **(D)** ovarian cancer cells SKOV3, **(E,F)** prostate cancer PC3 and DU145 cell lines were treated with varying concentrations of SK4 then cell growth inhibition was assessed through sulforhodamine B assays following two cell doublings. Growth inhibition was normalised to untreated control and results were analysed through Graphpad Prism version 9.0 four parameter non-linear regression. Error bars represent ± standard error of the means of at least three representative experiments.

**FIGURE 3:**
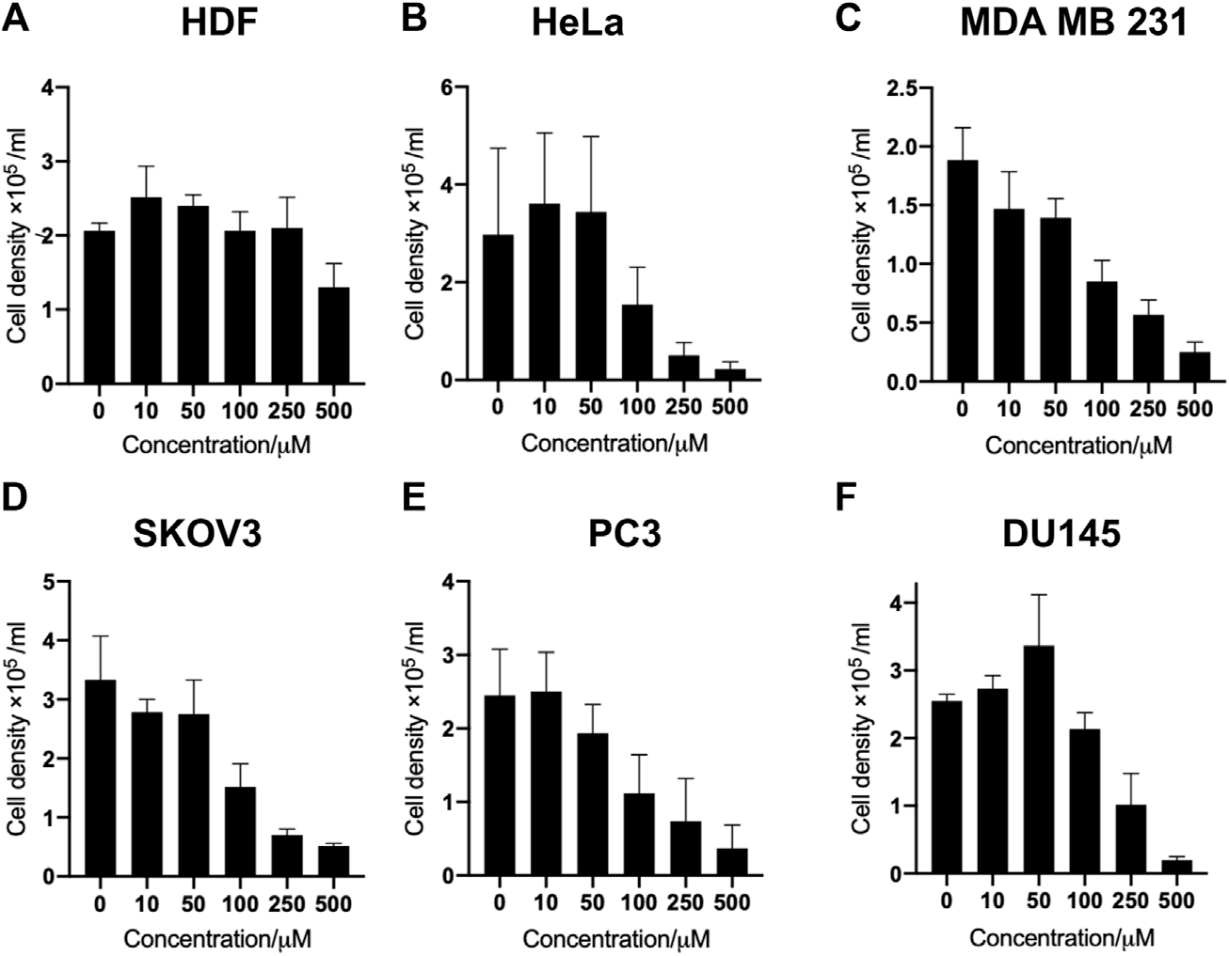
SK4 treatment induces loss of cell viability across panel of cell lines. **(A)** Human dermal fibroblasts, **(B)** cervical cancer HeLa, **(C)** breast cancer MDA MB 231, **(D)** ovarian cancer cells SKOV3, **(E,F)** prostate cancer PC3 and DU145 cell lines were treated with varying concentrations of SK4 then cell viability was assessed through trypan blue staining and counting on a haemocytometer slide. Error bars represent ± standard error of the means of at least three representative experiments.

**FIGURE 4:**
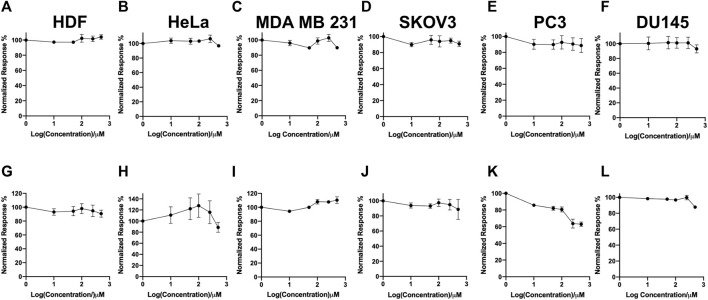
Control compounds demonstrate the iron chelation moiety is essential for inducing loss of cell viability. **(A,G)** Human dermal fibroblasts, **(B,H)** cervical cancer HeLa, **(C,I)** breast cancer MDA MB 231, **(F,L)** ovarian cancer cells SKOV3, **(D,E,K,L)** prostate cancer PC3 and DU145 cell lines were treated with varying concentrations of SK4C1 **(A–F)** or SK4C2 **(G–L)** then cell viability was assessed through sulforhodamine B assays. Error bars represent ± standard error of the means of at least three representative experiments.

In this study, a panel of cell lines from varying tissue origins were treated with increasing concentrations of SK4 to determine their sensitivity. Cell viability was determined through two assays: SRB assays to evaluate growth inhibition and trypan blue staining and counting. SK4 induced cytotoxicity in a dose-dependent manner (Figure 2). Cell lines displayed GI50s ranging from 111.3 µM to above 500 µM with the most sensitive cell line being HeLa cells (cervical cancer) and the least sensitive being the normal human dermal fibroblasts (NHDF) (Table 1). Prostate PC3 cell lines were more sensitive (GI50 = 117.4 µM) to SK4 compared to less invasive prostate DU145 cells (GI50 = 237.3 µM) (Figures 2E,F) (Table 1). Overall, the pattern of sensitivity was HeLa > PC3 > SKOV3 > DU145 > MDA MB 231 > HDF. Interestingly, IC50s obtained through cell count were lower than GI50s obtained through SRB assays, this could potentially indicate cell swelling (data not shown) (Figure 3) (Table 1). MDA MB 231 cell line (triple negative breast cancer) showed a GI50 of >500 µM in comparison to an IC50 of 316 µM obtained from trypan blue staining (Table 1). For further experiments to characterize SK4 mechanism of action, MDA MB 231 cells were selected as a cancer cell line with a high IC50 and SKOV3 (ovarian cancer) were selected as a cancer cell line with a low-intermediate IC50. To validate SK4 structure activity we used control compounds SK4C1 and SK4C2 each lacking a key moiety, with SK4C1 lacking the iron chelation moiety and SK4C2 lacking the amino acid moiety which allows entry through LAT1 (Figures 1B,C). Viability assays with SK4C1 indicated no change in cell viability whereas a slight decline in cell viability was detected at the higher concentrations of SK4C2, thus confirming cytotoxicity is driven by the iron chelation moiety (Figure 4).

**TABLE 1:** Average GI50 obtained through sulforhodamine B assays and IC50 obtained through trypan blue count of panel of cell lines ± standard error.

Cell line	GI50 (µM)	IC50(µM)
HeLa	111.3 ± 12.2	98.3 ± 12.4
HDF	>500	>500
DU145	237.3 ± 7.4	186.6 ± 60.2
PC3	117.4 ± 6.6	77.3 ± 76.6
SKOV3	146.5 ± 1.3	87.2 ± 18.3
MDA MB 231	>500	316.0 ± 24.2

### 3.2 SK4 drives apoptosis in MDA MB 231 cells

**FIGURE 5:**
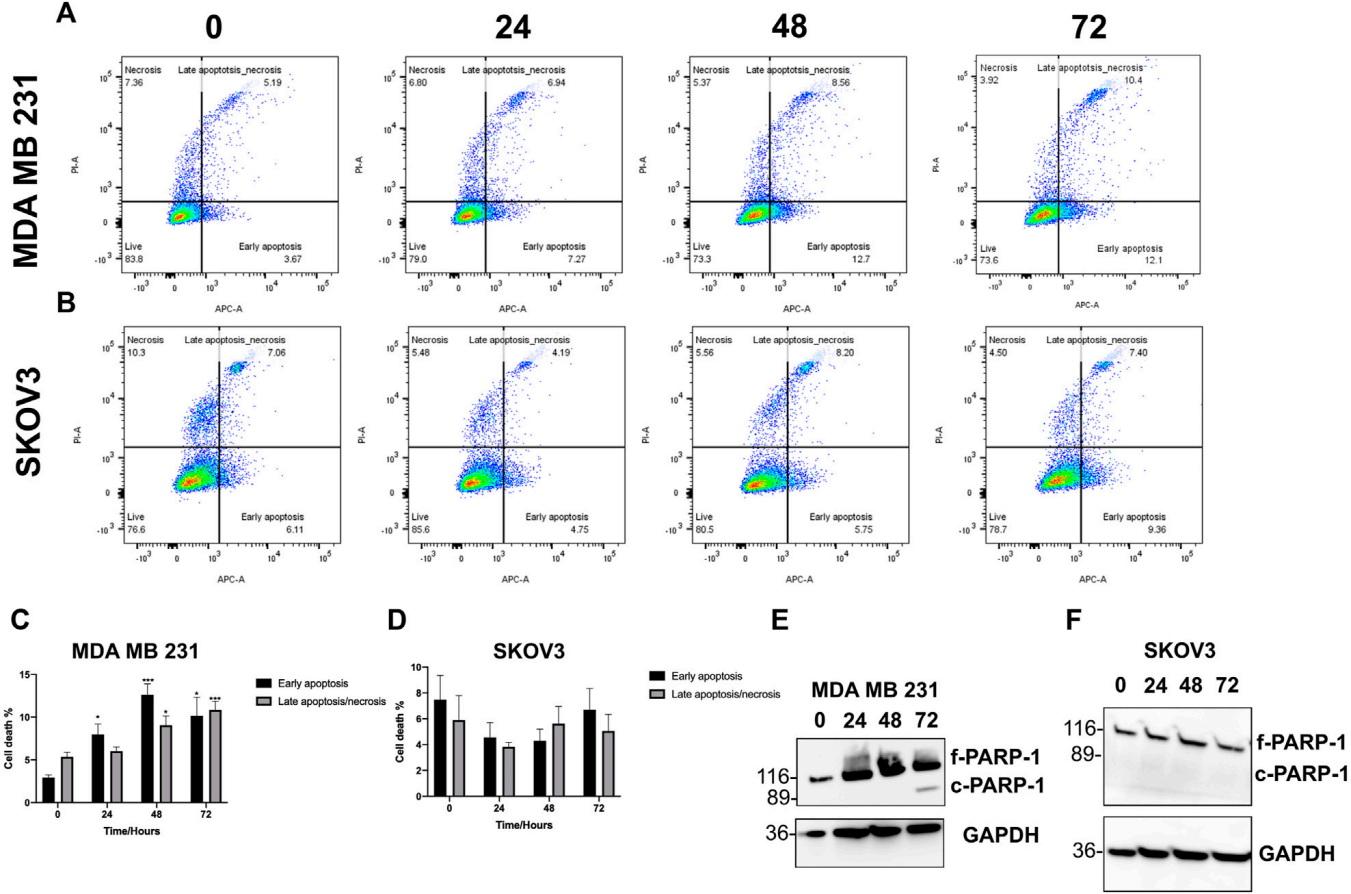
MDA MB 231 undergo apoptosis in response to treatment with SK4. **(A,C)** MDA MB 231 and **(B,D)** SKOV3 cell lines were treated with SK4 then stained with annexin V and propidium iodide staining and flow cytometry with analysis on FlowJo™ v10.8 Software. Western blots were conducted on **(E)** MDA MB 231 and **(F)** SKOV3 treated cell lysates to assess PARP-1 cleavage. Immunoblots are representative of three independent experiments. Error bars represent ± standard error of the mean of three independent experimental replicates. Significance is indicated as follows: *p < 0.05, **p < 0.005, ***p < 0.0005.

Following cell viability experiments, MDA MB 231 and SKOV3 were treated with their respective IC50/GI50 dose of SK4 for 24, 48 and 72 h and assessed for apoptotic induction through PARP-1 cleavage and Annexin V assays. MDA MB 231 cells underwent early apoptosis at 24 h then late apoptosis/necrosis at 48 and 72 h (Figures 5A,C). PARP-1 cleavage was observed at 72 h confirming SK4 induction of apoptosis in MDA MB 231 cells (Figure 5E, Supplementary Figure S1). SKOV3 cells did not display signs of apoptosis at any timepoints suggesting a different mechanism of cell death is behind SK4 driven cytotoxicity in the SKOV3 cell line (Figures 5B,D,F, Supplementary Figure S2).

### 3.3 SK4 induces cell cycle arrest at S phase

**FIGURE 6:**
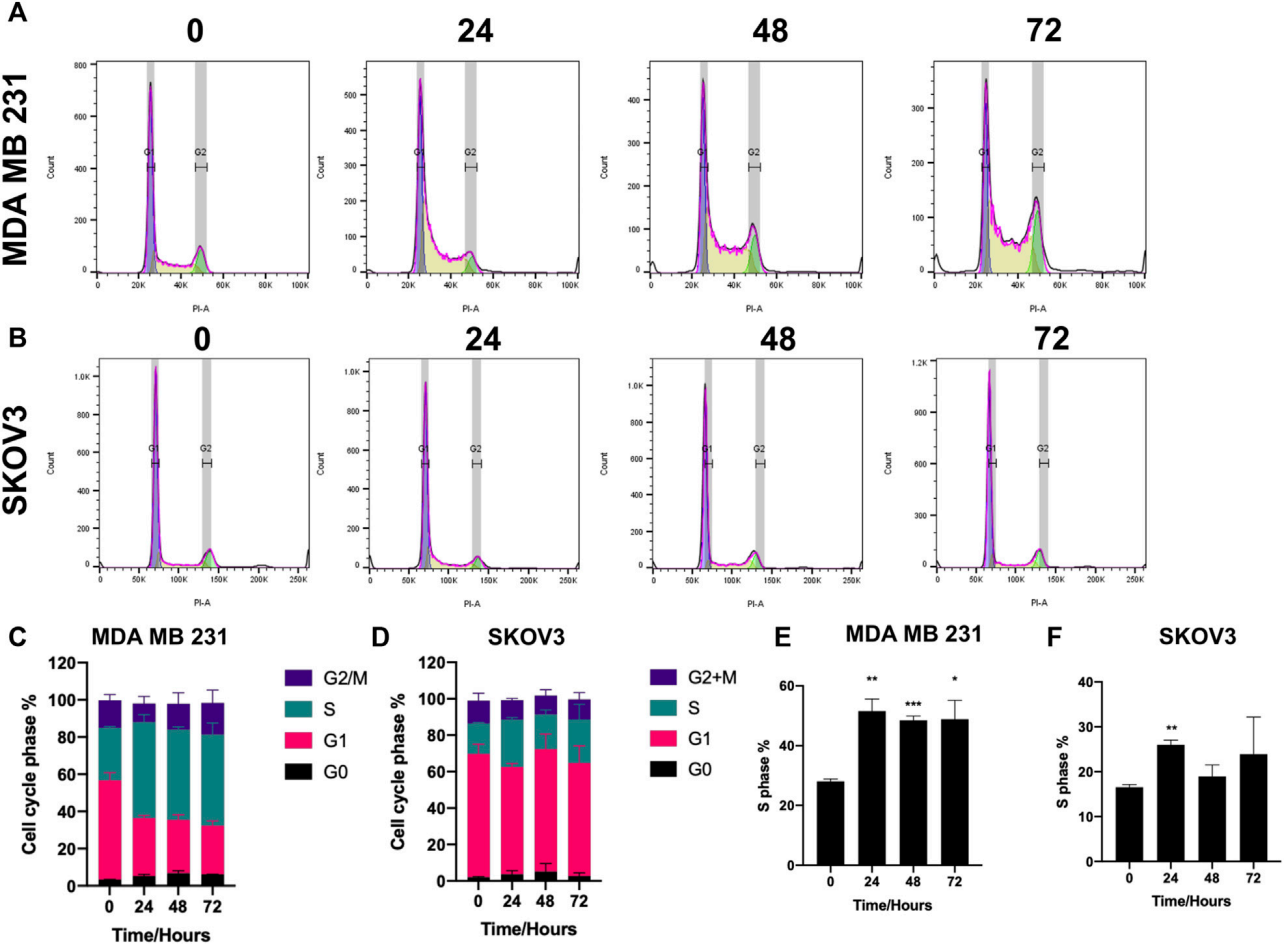
SK4 induces S phase arrest. **(A,C,E)** MDA MB 231 and **(B,D,F)** SKOV3 cells were treated with their respective IC50 and GI50 of SK4 for a timecourse. Cells were fixed stained with propidium iodide and cell cycle distribution was assessed by flow cytometry and analysed by FlowJo™ v10.8 Software. Error bars represent ± standard error of the mean of three independent experimental replicates. Significance is indicated as follows: *p < 0.05, **p < 0.005, ***p < 0.0005.

Cell cycle analysis was conducted using propidium iodide staining and flow cytometry to elucidate the effect of SK4 on the cell cycle. MDA MB 231 cell cycle distribution was impacted by SK4 treatment, with a decrease in cells at G1 and an increase of cells at S phase consistent with S phase arrest, which was observed at 24, 48 and 72 h (Figures 6A,C,E). SKOV3 cell lines showed significant S phase arrest at 24 h which reverted to normal cell cycle distribution at 48 and 72 h (Figures 6B,D,F).

### 3.4 SK4 upregulates tumor suppressor gene NDRG1

**FIGURE 7:**
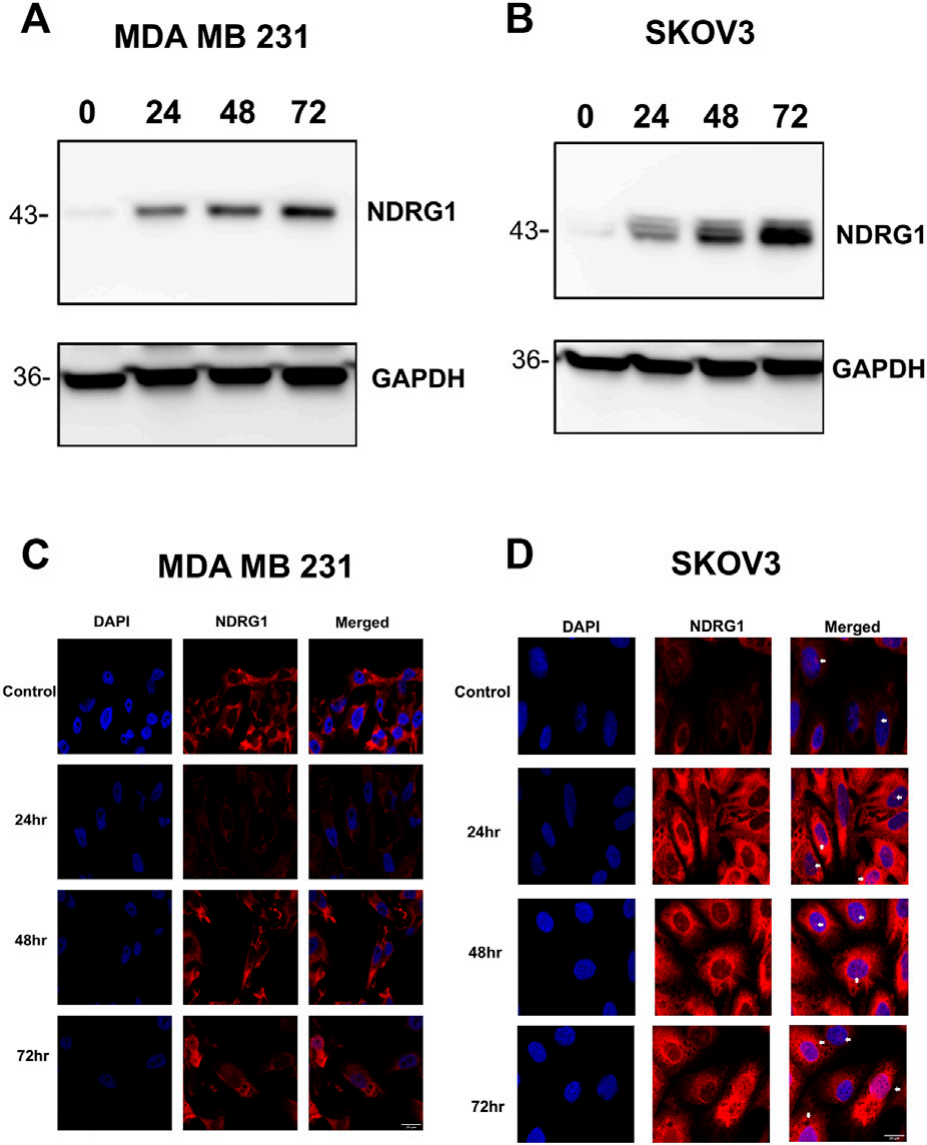
Tumour suppressor NDRG1 is upregulated in response to SK4. Treated **(A)** MDA MB 231 and **(B)** SKOV3 cell lines were collected and analysed with western blotting for NDRG1 expression. Immunofluorescence was performed on treated **(C)** MDA MB 231 and **(D)** SKOV3 cells to assess NDRG1 localisation. Immunoblots are representative of three independent experiments. Magnification ×40. Scale bar = 20 µM.

Previous studies have implicated tumor suppressor NDRG1 in mediating iron chelation mechanism of action through the inhibition as EGFR, TGF-β, and STAT3 (Le and Richardson, 2004; Lui et al., 2015b; Menezes et al., 2019; Yang et al., 2021). Treating MDA MB 231 and SKOV3 cells with SK4 resulted in a time-dependent upregulation of NDRG1 protein expression (Figures 7A,B, Supplementary Figure S3). Immunofluorescence demonstrated differential NDRG1 localization in each cell line, with MDA MB 231 basal localization concentrated in the cytoplasm, and nuclear localization was only observed at the 72 h timepoint (Figure 7C). In SKOV3 cells NDRG1 concentrated in the nucleus and cytoplasm basally and after treatment with SK4 (Figure 7D). This implies despite significant upregulation of NDRG1 in both cell lines, NDRG1 may perform different roles in SKOV3 and MDA MB 231 cell lines basally and in response to treatment. As NDRG1 upregulation has been implicated in suppression of migration, scratch wound assays were performed to investigate the impact of SK4 on cell migration (Lui et al., 2015b). SK4 was found to have no impact on cell migration in SKOV3 and MDA MB 231 cell lines (Supplementary Figures S4, S5).

### 3.5 Knockdown of NDRG1 impacts MDA MB 231 sensitivity to SK4 but not SKOV3

**FIGURE 8:**
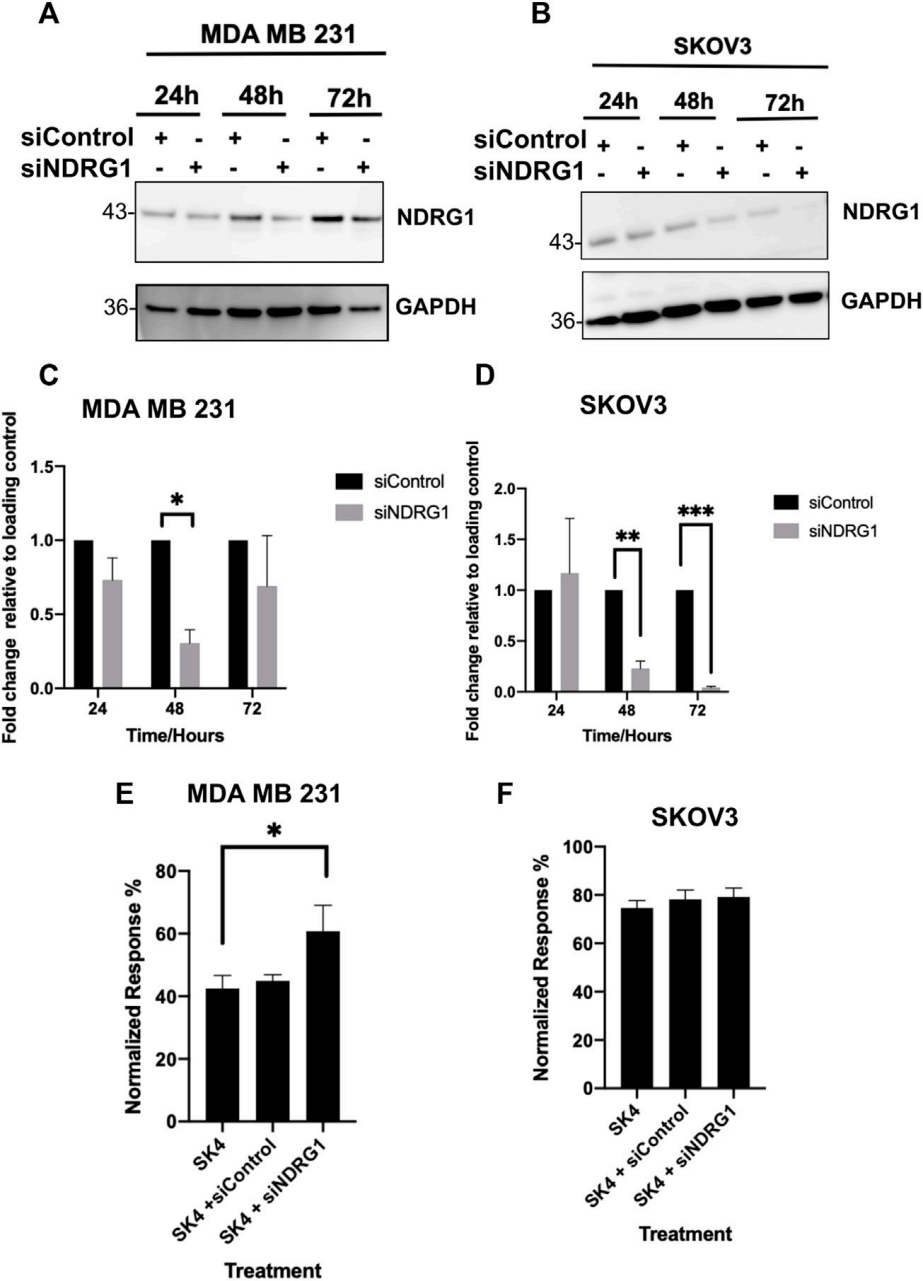
Loss of NDRG1 expression antagonises SK4 activity in MDA MB 231. **(A,C)** MDA MB 231 and **(B,D)** SKOV3 cell lines were incubated with Control and NDRG1 siRNAs and cell lysates were harvest and assessed through western blotting **(A–D)** or combined with SK4 and cell viability was analysed **(E,F)**. Immunoblots are representative of three independent experiments. Error bars represent ± standard error of the mean of three independent experimental replicates. Significance is indicated as follows: *p < 0.05, **p < 0.005, ***p < 0.0005.

To determine if NDRG1 was involved in SK4 driven cytotoxicity, cells were treated with control siRNA and NDRG1 siRNA alone and in combination with SK4 (Figure 8, Supplementary Figure S6). Interestingly, knockdown of NDRG1 decreased cell viability MDA MB 231 cells, but not in SKOV3 cells (Data not shown). When the treated cells were normalized for their respective controls, NDRG1 knockdown was shown to antagonize SK4 treatment in MDA MB 231 cells but had no impact on SKOV3 sensitivity to SK4 (Figure 8). This corresponds to NDRG1 pleiotropy and suggests SK4 mediates its role *via* NDRG1.

## 4 Discussion

Tumors of varying origins adapt their iron metabolism to maximize their proliferation. Breast, prostate, and colorectal cancers display an upregulation of iron import proteins and a downregulation of iron export proteins (Cui et al., 2007; Zhang et al., 2020; Wei et al., 2021). “Iron ad-diction” represents a potential target for cancer therapy which warrants further investigation as to its mechanisms of action and improvement of its selectivity. Our group has designed SK4 which has shown promising results in melanoma *in vitro* (Kyriakou et al., 2020; Kyriakou et al., 2021). In this paper, we present an NCBI approved screen of SK4 resulting in a broad range of sensitivities in different cell lines, with HeLa cells being the most sensitive and normal primary cells HDFs displaying limited sensitivity. Additionally, structural functional studies have validated the iron chelation moiety as being responsible for inducing cytotoxicity.

SK4 represents is the first iron chelator designed to gain cell entry through the LAT1 transporter exploiting “iron addiction” as well as dysregulated amino acid metabolism. LAT1 overexpression has been associated with poorer patient prognosis, and metastatic tumors, thus limiting iron chelation entry to the LAT1 transporter could be more suited to malignant tumors and could dampen potential side effects associated with iron chelation such as methemoglobinemia and myelosuppression (Yu et al., 2012a; Ichinoe et al., 2015; Ichinoe et al., 2021; Kurozumi et al., 2022). Interestingly, LAT1 is an antiport so while it can allow the entry of amino acids it has no effect on the total amount of amino acids within the cells but can influence the proportions of different amino acids, so SK4 may compete with amino acids for cell entry (Yanagida et al., 2001; Verrey et al., 2004). Our control compound studies with SK4C1, which lacks the iron chelation moiety prove SK4 mediated cytotoxicity is reliant on the iron chelation moiety for cytotoxicity, therefore the decline in cell viability is due to iron chelation not competition with amino acids. Additionally, entry through the LAT1 transporter is essential for cell targeting; control compound SK4C2 which lacks the amino acid moiety, induced limited cytotoxicity at the highest dose.

Iron chelation by SK4 has demonstrated its ability to inducing apoptosis in melanoma (Kyriakou et al., 2020). Studies have shown iron chelation induces apoptosis in a p53-independent manner making it a suitable therapy for p53 deficient tumors, which comprise 50% of all tumors (Tian et al., 2010; Bailey et al., 2018; Schaafsma et al., 2022). No apoptotic markers were observed in the SKOV3 cell line implying another mechanism of cell death is responsible for SK4 mediated cytotoxicity. Iron chelation has been observed to induce autophagy and other mechanisms of cell death such as mitophagy and a methuosis-like cell death (Sahni et al., 2014; De Bortoli et al., 2018; Si et al., 2020). SK4 also induced S phase arrest in MDA MB 231 cells which was sustained and SKOV3 cells which returned to their normal cell cycle distribution at 48 h of treatment. Iron chelation has been shown to prevent ribonucleotide reductase action hence inhibiting DNA synthesis which occurs at S phase, triggering G1/S phase arrest (Nyholm et al., 1993; Yu et al., 2011). Iron chelator L-mimosine induces S phase arrest in HeLa cells after the cells had initiated DNA synthesis, suggesting L-mimosine analogue SK4 may have a similar mechanism of action (Hughes and Cook, 1996). Additionally, iron chelation inhibits expression of c-myc and cyclin D1 cell cycle proteins which in-duce S phase entry (Nurtjahja-Tjendraputra et al., 2007). Additionally, many cell cycle proteins are iron dependent such as CDK1 (Kuang et al., 2019).

Tumor suppressor NDRG1 has been strongly linked to iron chelation mediated cytotoxicity. Iron chelation upregulates NDRG1 which in turn inhibits signaling pathways such as EGFR, TGF- β, and STAT3 (Le and Richardson, 2004; Lui et al., 2015b; Menezes et al., 2019; Yang et al., 2021). SK4 has been shown to upregulate NDRG1 protein expression in MDA MB 231 cell line and SKOV3 in a time dependent manner. Studies with MDA MB 231 cell line have shown NDRG1 can upregulate some oncogenic pathways, such as PI3K signaling, suggesting NDRG1 pleiotropy (Chen et al., 2019). NDRG1 has been shown to be involved in lipid metabolism in breast cancer cell lines, aiding in cell survival in lipid poor environments (Sevinsky et al., 2018).

Interestingly, NDRG1 showed differential localization in MDA MB 231 cells and SKOV3 cells with basal expression of NDRG1 concentrated to the cytoplasm in MDA MB 231 cell line in contrast SKOV3 cells had expression in the nucleus and cytoplasm. Treatment with SK4 induced nuclear localization in the MDA MB 231 cells at the 72-h timepoint and no change in NDRG1 localization with the SKOV3 cells. NDRG1 nuclear localization coincides with PARP-1 cleavage in the MDA MB 231 cells. NDRG1 has been shown to localize in the nucleus following treatment with iron chelators and DNA damaging agents, potentially basal nuclear localization could be a resistance mechanism against iron chelator mediated apoptosis (Kurdistani et al., 1998; Park et al., 2018). Differential NDRG1 localization has been observed depending on the tissue type with nuclear localization more common in PTEN wildtype cells. MDA MB 231 and SKOV3 cells both have wildtype PTEN suggesting that other factors could be involved in localization. NDRG1 has been determined not to be a transcriptional factor as it lacks the correct sequence, but it does carry out some important functions within the nucleus (Park et al., 2018). NDRG1 stabilizes O^6^-methylguanine-DNA methyl-transferase for DNA damage repair (Weiler et al., 2014). Additionally, NDRG1 can interact with the DNA polymerase clamp in Kaposi’s sarcoma-associated herpesvirus infected cells (Zhang et al., 2019). This implies a potential role of NDRG1 in response to replication stress induced through loss of ribonucleotide activity.

NDRG1 has been revealed to act as a tumor suppressor and an oncogene depending on the tumor stage, site of origin and molecular subtype. MDA MB 231 cells responded to knockdown by upregulating NDRG1 expression at 72 h whereas as the knockdown was more stable with the SKOV3 cells. MDA MB 231 are more sensitive to loss of NDRG1 yet knockdown of NDRG1 antagonizes SK4, this indicates NDRG1 is involved in pro-survival and pro-death pathways. *In vitro* work in ovarian cancer has shown NDRG1 knockdown increases cell proliferation and inhibits apoptosis suggesting a tumour suppressor role in ovarian cancer (Wang et al., 2014). In contrast, high levels of NDRG1 has been associated with poor prognosis in breast cancer and NDRG1 is a biomarker for aggressive breast cancer, suggesting a oncogenic role in breast cancer (Sevinsky et al., 2018; Villodre et al., 2020). A meta-analysis on the correlation between NDRG1 and patient prognosis demonstrated NDRG1’s impact on prognosis is dependent on the cancer type; low NDRG1 expression is correlated to poor overall survival in colorectal cancer but improved overall survival in liver cancer and had no impact in gastric cancer. During apoptosis p53 induces NDRG1 gene expression and p53 dependent apoptosis is reliant on NDRG1 expression (Stein et al., 2004). The level expression of NDRG1 in the tissue of origin may influence NDRG1’s role during carcinogenesis, for example a high level of NDRG1 implies an active role in cell maintenance, so loss of expression could drive carcinogenesis (Chen et al., 2018). A truncated isoform of NDRG1 has been observed in prostate cancer cell lines but not normal cell lines, this could explain the contradictory roles observed in cells, the full length isoform may be responsible for the tumour suppressor roles of NDRG1 and the truncated version for the oncogenic roles, this warrants further investigation (Park et al., 2018). In addition to NDRG1 upregulation, iron chelation can inhibit cancer cell stemness (Szymonik et al., 2021). Cancer stem cells present a roadblock to full patient recovery as their capacity for self-renewal can fuel metastasis and drug resistance (Yu et al., 2012b; Szymonik et al., 2021). Chemotherapeutic drugs fluorouracil and cisplatin inhibited the proliferation of cancer stem cells but failed at altering the expression of stemness markers, whilst iron chelator DFO decreased the expression of Nanog, Oct3/4 and c-Myc *in vivo* and *in vitro* (Ninomiya et al., 2017).

In summary, we have presented novel iron chelator SK4, which utilizes the LAT1 amino acid transporter as its route of cell entry. We have investigated SK4-mediated cytotoxicity across a panel of tumor-derived cell lines through our NCBI approved screen. We have also elaborated on SK4 mechanism of action in ovarian and triple negative breast cancer cells in which we see differential modes of cell death and involvment of tumour suppressor NDRG1. SK4 will be used as a starting point for the creation of more iron chelation compounds which enter through LAT1 exerting a double pronged approach of targeting “iron addiction” to induce cytotoxicity and dysregulated amino acid metabolism across a range of tumor derived cell lines although the mechanism of cell death may vary.

## Data Availability

The raw data supporting the conclusion of this article will be made available by the authors, without undue reservation.
