# Supplementary material for: Novel iron chelator SK4 demonstrates cytotoxicity in a range of tumour derived cell lines
Source: Front Mol Biosci. 2022 Sep 23;9:1005092. doi: 10.3389/fmolb.2022.1005092 (PMC9540520; doi:10.3389/fmolb.2022.1005092)
Supplement: Supplementary file 1 [file DataSheet1.docx]

Supplementary Material





Supplementary Figure 1: Full length western blot for PARP-1 cleavage in MDA MB 231. * NDRG1 band remaining after reprobing with GAPDH





Supplementary Figure 2: Full length western blot for PARP-1 cleavage in SKOV3





Supplementary Figure 3: Full length NDRG1 western

**
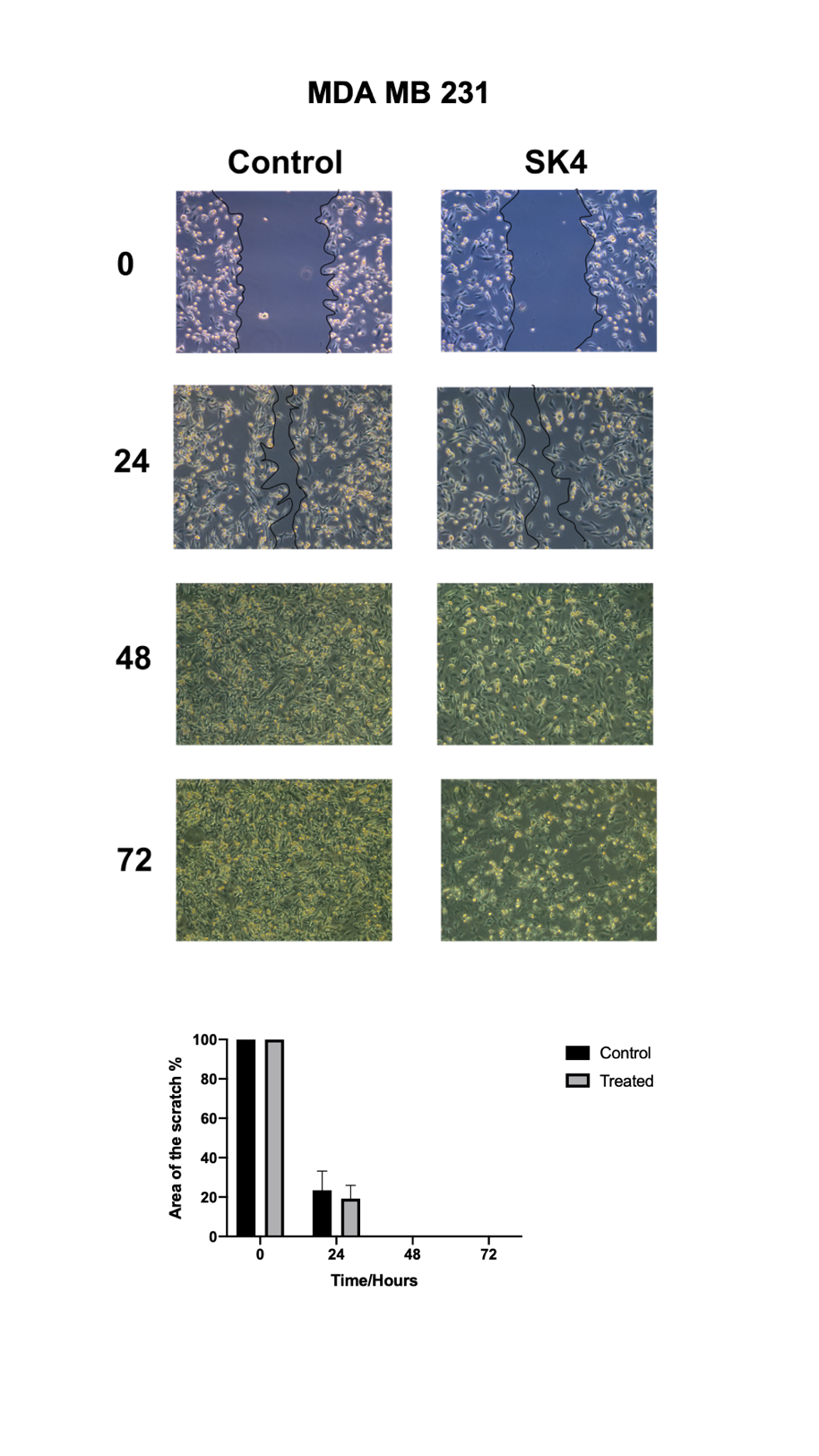
**

Supplementary Figure 4: MDA MB 231 scratch wound assay


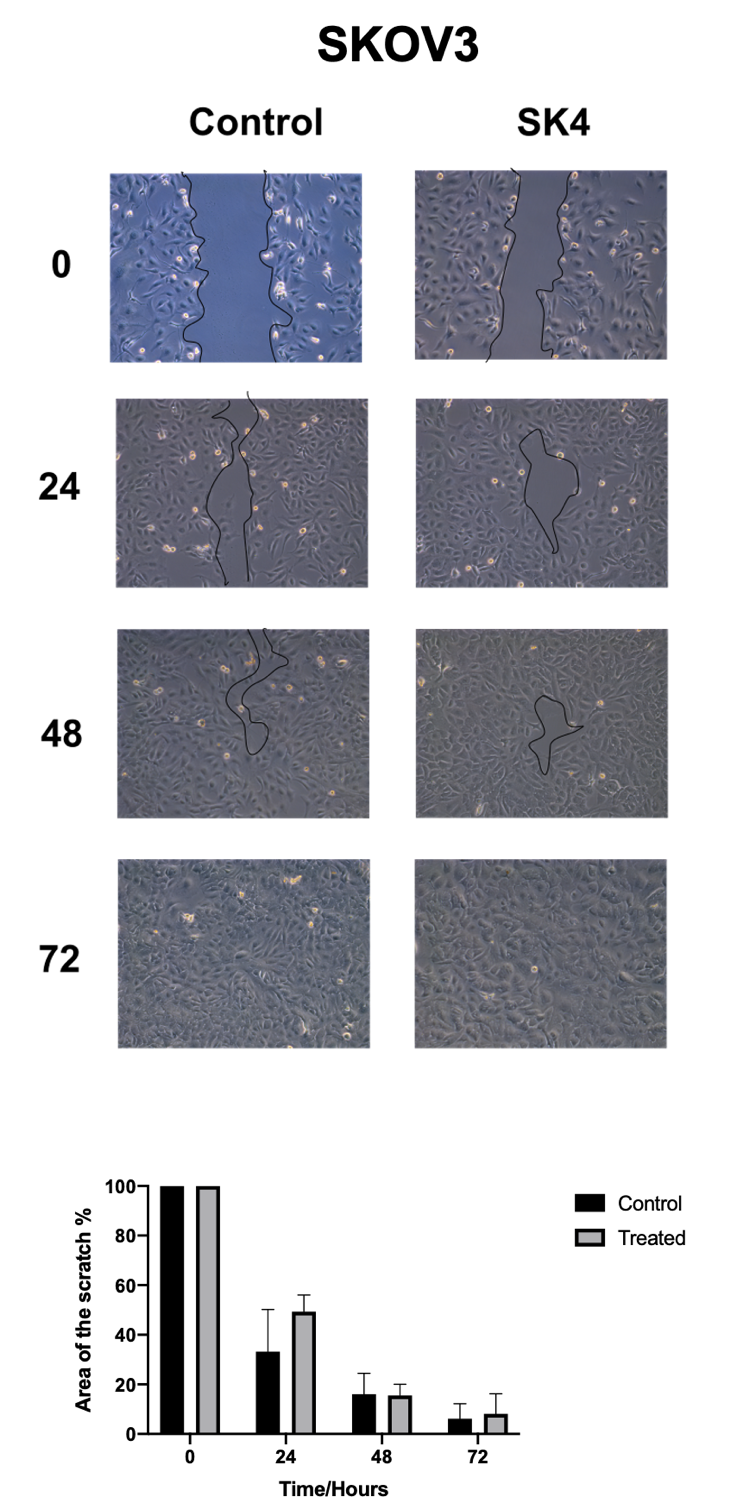


Supplementary Figure 5: SKOV3 scratch wound assay

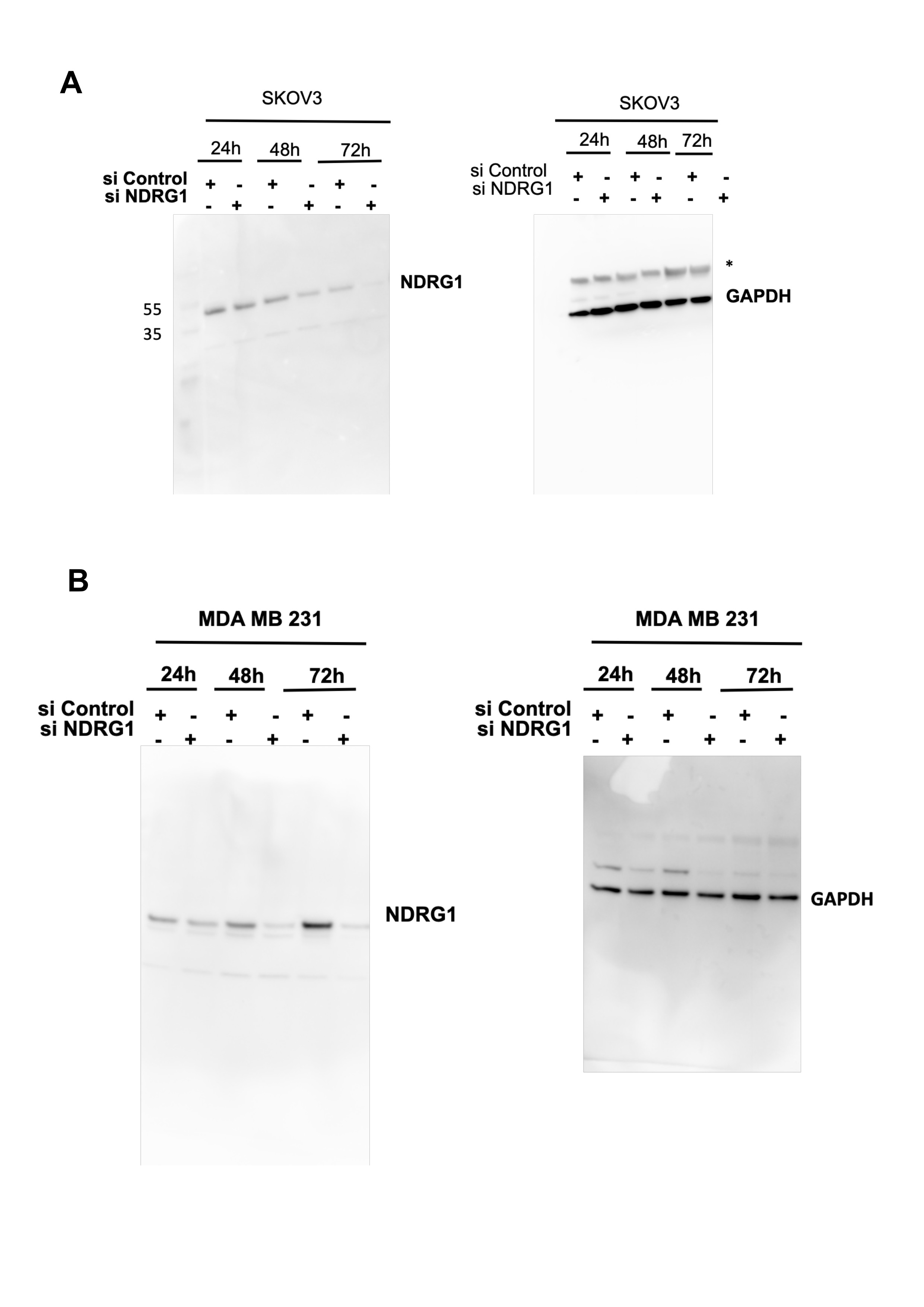


Supplementary Figure 6: Full length NDRG1 knockdown western blot
